# Trends in Prescription of Stimulants and Narcoleptic Drugs in Switzerland: Longitudinal Health Insurance Claims Analysis for the Years 2014-2021

**DOI:** 10.2196/53957

**Published:** 2025-01-07

**Authors:** Tamara Scharf, Carola A Huber, Markus Näpflin, Zhongxing Zhang, Ramin Khatami

**Affiliations:** 1 Graduate School of Health Sciences, University of Bern, Bern, Switzerland; 2 Institute of Primary Health Care (BIHAM), University of Bern, Bern, Switzerland; 3 Centre of Sleep Medicine and Epileptology Barmelweid, Klinik Barmelweid AG, Aargau, Switzerland; 4 Department of Health Sciences, Helsana Group, Zürich, Switzerland; 5 Institute of Primary Care, University of Zürich, University Hospital Zürich, Zürich, Switzerland; 6 Barmelweid Academy, Klinik Barmelweid AG, Barmelweid, Switzerland; 7 Department of Neurology, Inselspital Bern University Hospital, University of Bern, Bern, Switzerland

**Keywords:** prescription trends, claims data, cross-sectional data, narcolepsy, prescribers, prescribing practices, medical care, stimulants, stimulant medication

## Abstract

**Background:**

Stimulants are potent treatments for central hypersomnolence disorders or attention-deficit/hyperactivity disorders/attention deficit disorders but concerns have been raised about their potential negative consequences and their increasing prescription rates.

**Objective:**

We aimed to describe stimulant prescription trends in Switzerland from 2014 to 2021. Second, we aimed to analyze the characteristics of individuals who received stimulant prescriptions in 2021 and investigate the link between stimulant prescriptions and hospitalization rates in 2021, using hospitalization as a potential indicator of adverse health outcomes.

**Methods:**

Longitudinal and cross-sectional data from a large Swiss health care insurance were analyzed from all insureds older than 6 years. The results were extrapolated to the Swiss general population. We identified prescriptions for methylphenidate, lisdexamfetamine, modafinil, and sodium oxybate and calculated prevalences of each drug prescription over the period from 2014 to 2021. For 2021 we provide detailed information on the prescribers and evaluate the association of stimulant prescription and the number and duration of hospitalization using logistic regression models.

**Results:**

We observed increasing prescription rates of all stimulants in all age groups from 2014 to 2021 (0.55% to 0.81%, 43,848 to 66,113 insureds with a prescription). In 2021, 37.1% (28,057 prescriptions) of the medications were prescribed by psychiatrists, followed by 36.1% (n=27,323) prescribed by general practitioners and 1% (n=748) by neurologists. Only sodium oxybate, which is highly specific for narcolepsy treatment, was most frequently prescribed by neurologists (27.8%, 37 prescriptions). Comorbid psychiatric disorders were common in patients receiving stimulants. Patients hospitalized in a psychiatric institution were 5.3 times (odds ratio 5.3, 95% CI 4.63‐6.08, *P*<.001) more likely to have a stimulant prescription than those without hospitalization. There were no significant associations between stimulant prescription and the total length of inpatient stay (odds ratio 1, 95% CI 1‐1, *P*=.13).

**Conclusions:**

The prescription of stimulant medication in Switzerland increased slightly but continuously over years, but at lower rates compared to the estimated prevalence of central hypersomnolence disorders and attention-deficit/hyperactivity disorders/attention deficit disorders. Most stimulants are prescribed by psychiatrists, closely followed by general practitioners. The increased odds for hospitalization to psychiatric institutions for stimulant receivers reflects the severity of disease and the higher psychiatric comorbidities in these patients.

## Introduction

Stimulants such as methylphenidate, lisdexamfetamine, and modafinil are highly potent pharmacologic treatment options for hypersomnolence disorders, including narcolepsy or attention-deficit/hyperactivity disorders/attention deficit disorders (ADHDs/ADDs).

ADHDs/ADDs are some of the most common diagnosed psychiatric disorders worldwide with a prevalence of 5.3% worldwide in the years of 1978 to 2010 among people aged 18 years or younger [1]. Prevalence can be different due to varying diagnostic methods per country. In Switzerland, a prevalence of 5.2% was found in children aged 7 to 17 years and in adult men a prevalence of 4% [2,3].

Prescriptions of stimulant drugs have been increasing at various rates over the past decades, with methylphenidate showing an 8.2 fold increase from 1996 to 2013 [4,5]. In the United States, amphetamine and methylphenidate increased from 5.6% to 6.1% in adults aged 20 years or older between 2014 and 2019 [4,5]. In New Zealand, 1.06% of adolescents received stimulants in 2016, an increase of 41.3% from 0.75% in 2011 [6]. Denmark observed a trend in stimulant prescriptions rising from 0.31 per 1000 person-years in 1996 to 7.29 per 1000 person-years in 2010 [7]. In Switzerland, data on stimulant prescription rates is scarce. A Swiss study from 2015 found a lifetime prevalence of stimulants and other substances enhancing cognitive abilities of about 1.4% in employees and students [8]. No sufficient data have, however, been collected in a nationwide study.

Stimulant medication is used for diseases often manifesting during childhood or adolescence and in many cases long-term pharmacological treatment throughout adulthood is needed. Prescription of stimulant agents in this age group is therefore of special interest to balance the need of medication and potential risk by over prescription or under prescription. Prescription of stimulants in young age groups increased from 0.02% to 0.26% over time in Asia, Australia, Europe, and North America in children and 0.003% to 1.48% in adults [9]. Among Swiss school children between 2002 and 2005, methylphenidate prescription increased from 0.74% to 1.02% for children aged 5 to 14 years [10].

This rapid prescription increase may indicate over prescription or even misuse. Misuse of stimulants is common, with up to 17% prevalence in US college students according to a meta-analysis. Misuse can lead to a range of negative consequences such as decreased appetite, insomnia, and increases in heart rate and blood pressure with increased long-term cardiovascular risk and possibly lead to increased hospitalization rates [11-13].

Properly identifying current stimulant prescription rates and discovering the prescription patterns or circumstances of their prescription (prescriber, package size, and comorbidities) may help to further identify alarming prescription increases and potential misuse in Switzerland, and the possible causes.

We therefore had 2 objectives: first, we aim to describe the rate of stimulant prescriptions in Switzerland from 2014 to 2021, focusing on both minors and adults. We hypothesize that the rate of stimulant prescriptions in Switzerland, similar to international trends, has increased over the last decade, with the highest prescription rates being recorded in 2021.

Second, we aimed at analyzing the characteristics of individuals who received stimulant prescriptions in 2021. This analysis will include factors such as comorbidities, age, and prescription details such as package size and health care providers most frequently issuing these prescriptions. Our hypotheses are that recipients likely mirror the characteristics of ADHDs/ADDs and central hypersomnolence disorders, most are minors with few comorbidities, and the likelihood of receiving a prescription decreases with age. Specialists, particularly psychiatrists and neurologists, are expected to be the primary prescribers.

Lastly, this study aims to investigate the link between stimulant prescriptions and hospitalization rates in 2021, using hospitalization as a potential indicator of adverse health outcomes. Given the expected increase in stimulant prescriptions until 2021, the hypothesis is that individuals prescribed stimulants may have a higher risk of hospitalization, particularly if prescription rates are on the rise.

## Methods

### Study Design

This study is a longitudinal and cross-sectional analysis of the Helsana health care insurance data of around 1.5 million people in Switzerland insured over the period of 2014 to 2021. Helsana belongs to a group of the biggest insurance companies in Switzerland and insures 14% of the Swiss population, with insureds in 26/26 cantons of Switzerland. Data describes general information on the insured persons and all their invoices for health services directed to the insurance. These invoices are representative of all health care costs of the insureds, except for the costs that were not sent as invoices to the insurance and paid by the insureds themselves (ie, over the counter drug costs and dental costs). We decided to provide more detailed descriptive statistics for data in 2021, since it was the most recent data available to us and presumably with the highest rate of stimulant prescription.

### Identification of Drugs

We identified the drug invoices through their Anatomical Therapeutic Chemical code, which classifies chemical substances based on their therapeutical properties. The identification was performed for the following drugs: methylphenidate (N06BA04), lisdexamfetamine (N06BA12), modafinil (N06BA07), and sodium oxybate (N07XX04) which is a specific medication for narcolepsy treatment and used to estimate the treatment prevalence of narcolepsy patients in the dataset. Pitolisant (N07XX11) was identified too as a stimulant with specific use for narcolepsy but was excluded from further analysis, as it was only authorized for use in 2020 but had neglectable low prescription rates. We also identified the Swissmedic code of the medications, which is specific not only for the chemical substance but also the producer of the medication and package size. These drugs are only accessible through prescription by a medical professional and reimbursed by the insurance company. Overlapping prescriptions of the 4 drugs was defined as 1 prescription of one of the 4 drugs invoiced with at least one of the other drugs once or multiple times during the year of 2021.

### Variables

The dataset consisted of all insureds aged ≥6 years with information on their age, sex, region of language, and region of residence. We categorized 5 age groups (6‐17 y, 18‐35 y, 36‐65 y, 66‐75 y, and 76+ y). We divided the insureds` residential regions into “rural,” “intermediate,” and “urban” subgroups according to the Swiss federal office for statistics.

The insureds had various health care plans including standard care and managed care models (eg family physician model). These health insurance plans were identified and categorized into standard care and managed care (ie, the combination of telemedical care and general practitioner [GP] care).

Chronic health condition status was identified by substance prescriptions related to chronic diseases. This was carried out according to approaches developed in previous research on the dataset [14]. We classified 22 different chronic conditions and categorized them into psychiatric, cardiologic, rheumatologic, respiratory comorbidities, or all other.

All invoices for hospitalization (ie, hospitals of all sizes providing acute care and psychiatric clinics) and the length of stay were included in our analysis.

Based on the medical prescriber who issued the invoices, several prescriber categories were defined: GP, psychiatrist, neurologist, other specialists (combining all other prescribers, such as nonspecific group practices, cardiologists, pulmonologists, rheumatologists, etc). Only health care personnel in Switzerland are allowed to prescribe medication. We further grouped them into “only prescriber” of the medication when a describer prescribed all medication exclusively for single individuals, “>50% prescriber” meaning more than 50% of the prescriptions for single individuals were invoiced by the prescriber, and “rest” with all other prescription proportions.

Within the prescriber categories (inpatient psychiatry, inpatient acute, and rehabilitation, nursing) we differentiated between inpatient (during a hospital stay) and outpatient invoices.

### Statistical Analysis

For our first objective we provide descriptive statistics for prescription trends among different age groups across the years 2014 and 2021 by identifying individuals in the dataset with at least one prescription of the predefined drugs. To obtain representative data for Swiss population we extrapolated these data by current residency numbers and populations statistics of the Federal Statistical Office.

For our second objective we restricted the descriptive statistics to the year 2021 and provide detailed information on age, sex, region of residence, health insurance status, comorbidities, prescribers, and package size. Logistic regression models were performed to evaluate the association of at least one stimulant prescription versus no prescription (ie, the dependent variable is prescription yes or no), number of hospitalization, and length of stay, adjusted for age, sex, region of residence, health insurance status, and number of chronic diseases for the year 2021.

All data management, graphic generation, and analysis was performed with the statistics program R (version 4.2.1; R Foundation).

### Data Availability

The authors were permitted access to the data by collaboration with the insurance companies research team. The datasets generated and analyzed during this study are available from the corresponding author on reasonable request. AI was not used in any way in data generation, analysis, and presentation of results.

### Ethical Considerations

According to ethical and legal regulations in Switzerland no ethical approval or patient consent was needed for this study, as all data complied with privacy regulations and personal data protection, data was anonymized when presented to the research team. The Swiss Human Research Act (REQ-2017‐00280) did not apply to this project. The exploratory statistical analyses of the feasibility test complied with the Swiss Federal Law on data protection. All data were anonymized and deidentified prior to the performed analysis to protect the privacy of patients, physicians, and hospitals. According to the national ethical and legal regulation, an ethical approval was not needed because the data were pre-existing and deidentified. Since data was anonymized, no consent of patients was required.

## Results

### Trend of Stimulant Prescription Per Year

As baseline we refer to the prescription period in 2014. Between baseline and 2021, on average 14% of Swiss people were at any time insured with the Helsana Group.

In the year 2014, 0.55% (42,848 insureds) of insured people of any age received at least 1 stimulant agent (or stimulant prescription). This number increased up to 0.8% (66,113 insureds) in the year of 2021. The largest growing percentage of stimulus prescriptions were in the youngest age group with an increase of prescription of 0.6 percentage points (17,972, 1.8% to 24,982, 2.4%) between 2014 and 2021 (Table 1).

Between 2014 and 2021, 32,2418 packages of methylphenidate were the most prescribed stimulant, followed by 46,074 packages of lisdexamfetamine, 8797 packages of modafinil, and 3115 packages of sodium oxybate. We found a prescription increase of all identified medications in younger and middle age groups over the years of 2014 to 2021, extrapolated by the Swiss population. The increase in prescription was steady in age groups aged 36‐65 years, whereas in other age groups prescription stagnated from 2018 to 2020, with a steep increase from 2020 to 2021.

**Figure 1.**
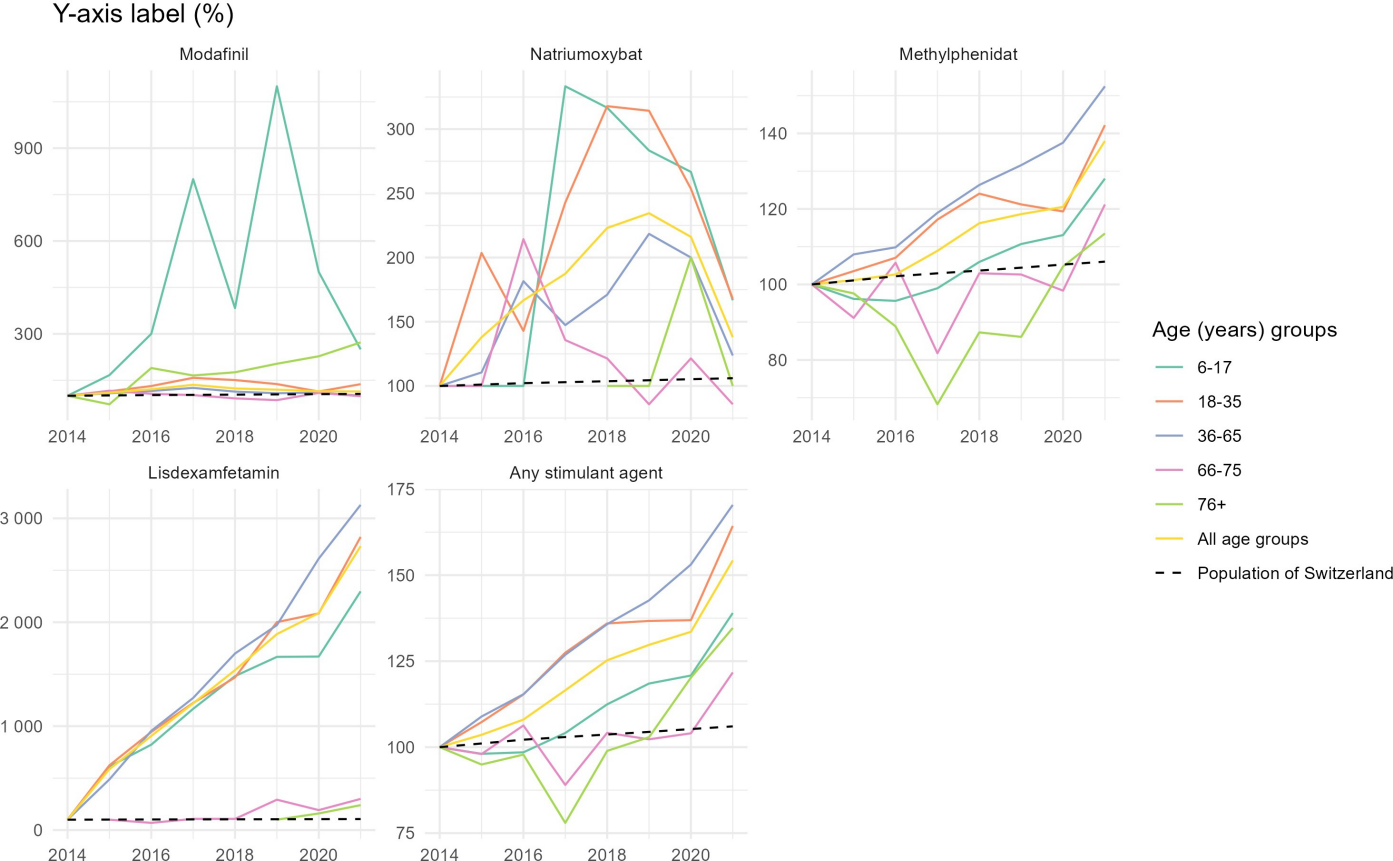
Trends of stimulant prescription from 2014 to 2021 per age group and active ingredient (indexed, base year=2014).

Methylphenidate prescription increased overall in all age groups with a steady increase in insureds aged 36‐65 years. All other age groups experienced a steep increase after 2020. Only insureds aged 66‐75 years and 76+ years ever experienced a smaller prescription rate than at baseline in 2014, with a drop to 89% (664/746) in 2017 (Figure 1).

Lisdexamfetamine prescription was low at baseline and increased steadily in all age groups with great increase from 0 prescriptions at baseline to 39 in insureds aged 60‐65 years. Smaller increase in prescription was seen in age groups aged 66‐75 years and 76+ years (Figure 1).

Prescription rates of modafinil—only prescribed to few—increased the most in the youngest age group (6‐17 y). The prescribing trend in this age group strongly fluctuated. From its highest peak in 2019 rates decreased from around 1100% (66/6) to slightly less than 300% (15/6) prescription compared to the baseline in 2014. In 2021, modafinil was most frequently prescribed in age groups aged 6‐17 years and 76+ years. All other age groups had only a moderate increase in modafinil prescriptions over the years 2014 to 2021.

Sodium oxybate overall increased the most in insureds aged 18‐35 years and 6‐17 years from 100% to 175% in 2021. Prescription rates for sodium oxybate only decreased overall in insureds aged 66‐75 years. Age groups aged 36‐65 years and 76+ experienced a similar prescription trend over the years with overall increase but declining prescription rates after 2020.

**Table 1.** Proportions of Swiss insureds with at least 1 stimulant agent or other narcolepsy treatment prescription within age groups for each year between 2014 and 2021.

Age (years)	2014, n (%)	2015, n (%)	2016, n (%)	2017, n (%)	2018, n (%)	2019, n (%)	2020, n (%)	2021, n (%)	*P* value (chi-square test for trend in proportions)
6‐17	17,972 (1.8)	17,624 (1.8)	17,702 (1.8)	18,711 (1.9)	20,209 (2)	21,296 (2.1)	21,714 (2.1)	24,982 (2.4)	<.001
18‐35	13,145 (0.68)	14,103 (0.72)	15,159 (0.77)	16,755 (0.85)	17,874 (0.91)	17,969 (0.92)	17,998 (0.92)	21,599 (1.10)	<.001
36‐65	10,708 (0.31)	11,661 (0.34)	12,355 (0.35)	13,584 (0.38)	14,537 (0.41)	15,277 (0.43)	16,394 (0.45)	18,251 (0.50)	<.001
66‐75	746 (0.1)	731 (0.1)	793 (0.1)	664 (0.1)	777 (0.1)	763 (0.1)	776 (0.1)	908 (0.1)	.10
76+	277 (0)	263 (0)	271 (0)	216 (0)	274 (0)	285 (0)	333 (0)	373 (0.10)	.05
All age groups	42,848 (0.55)	44,382 (0.57)	46,280 (0.59)	49,931 (0.63)	53,671 (0.67)	55,590 (0.69)	57,216 (0.70)	66,113 (0.81)	<.001

### Factors Associated With Prescription in 2021

#### Characteristics of Stimulant Users

Most stimulant receivers in the year of 2021 were male representing 61% (42,803/70,396) of our population. Only modafinil was more often prescribed to women than to men with 55% (970/1776) female receivers. The highest proportion of prescriptions was provided to people living in urban areas with a proportion of 67% (46,968/70,396) of all stimulants compared to intermediate and rural area residents. Managed care was the preferred health care plan for patients receiving stimulants with 72% (50,513/70,396) receiving. A total of 45% (798/1776) of all modafinil receivers had 3+ chronic illnesses, 55% (775/1399) of those had psychiatric comorbidities, followed by cardiological and rheumatological diseases. A total of 42% (49/121) of sodium oxybate users had no comorbidities. Most common chronic illness in sodium oxybate users was psychiatric (43/72, 60%) or cardiologic (31/72, 43%). Additionally, half of the methylphenidate users had comorbidities (28,619/57,128, 50%, 28,509 had no chronic illness identified) with psychological (14,396/28,619, 50%) and other chronic conditions (7034/28,619, 25%) as the most common identified chronic diseases. Similar results were found for lisdexamfetamine users, that is, 43% (4908/11,371) users had no comorbidities and psychological (3556/6463, 55%) and other (1989/6463, 31%) comorbidities were the most common chronic diseases (Table 2).

**Table 2.** Characteristics of Swiss insureds receiving any stimulant prescription in the year 2021.

Characteristic	Modafinil (n=1776)	Sodium oxybate (n=121)	Methylphenidate (n=57,128)	Lisdexamfetamine (n=11,371)
**Sex, n (%)**				
Male	806 (45.4)	63 (52.1)	35,038 (61.3)	6896 (60.7)
Female	970 (54.6)	58 (48)	22,090 (38.7)	4475 (39.4)
**Age (years)**				
Median (IQR)	45 (33-6)	38 (29-5)	22 (14-4)	26 (16-4)
Mean (SD)	46 (16)	40 (18)	27 (16)	28 (14)
**Age (years, in groups), n (%)**				
6‐17	16 (0.9)	10 (8.3)	22,948 (40.2)	3355 (29.5)
18‐35	568 (32)	47 (38.8)	18,002 (31.5)	4401 (38.7)
36‐65	976 (55)	48 (39.7)	15,157 (26.5)	3565 (31.4)
66‐75	139 (7.8)	12 (9.9)	742 (1.3)	38 (0.3)
76+	77 (4.3)	4 (3.3)	279 (0.5)	12 (0.1)
**Region of residence, n (%)**				
Urban	1060 (59.7)	80 (66.1)	38,196 (66.9)	7632 (67.1)
Intermediate	449 (25.3)	29 (24)	11,554 (20.2)	2422 (21.3)
Rural	267 (15)	12 (9.9)	7378 (12.9)	1317 (11.6)
**Health insurance status, n (%)**				
Managed care	1064 (59.9)	82 (67.8)	41,178 (72.1)	8189 (72)
Standard care	712 (40.1)	39 (32.2)	15,950 (27.9)	3182 (28)
**Comorbidities** [14]**, n (%)**				
0	377 (21.2)	49 (40.5)	28,509 (49.9)	4908 (43.2)
1	340 (19.1)	27 (22.3)	12,617 (22.1)	2601 (22.9)
2	261 (14.7)	25 (21.7)	6678 (11.7)	1619 (14.2)
3+	798 (44.9)	20 (16.5)	9324 (16.3)	2243 (19.7)
Most frequent, n (%)	psyd:^a^ 775 (43.6)	psyd: 43 (36)	psyd: 14,396 (25.2)	psyd: 3556 (31.3)
2nd most frequent, n (%)	card:^b^ 592 (33.3)	card: 31 (26)	ther:^c^ 7034 (12.3)	ther: 1989 (17.5)
3rd most frequent, n (%)	rheu:^d^ 430 (24.2)	rheu: 17 (14)	rheu: 6588 (11.5)	resp:^e^ 1435 (12.6)

a psyd: psychiatric.

b card: cardiological.

c ther: other.

d rheu: rheumatological.

e resp: respiratory.

#### Package Size

All medication was predominantly prescribed more than once within a year, with ≥5 packages prescribed in 40.4% (715/1768) of all modafinil prescriptions, 95.8% (115/120) of all sodium oxybate prescriptions, 47.5% (27,124/57,093) of all methylphenidate prescriptions, and 59.1% (6727/11,392) of all lisdexamfetamine prescriptions (Table 3).

**Table 3.** Number of Swiss insureds and their stimulant prescriptions by number of packages and by prescriber (profession of the physician) in the year 2021.

			At least 1 stimulant agent (total)	At least 1 modafinil use	At least 1 sodium oxybate use	At least 1 methylphenidate use	At least 1 lisdexamfetamin use
Total patients (N)			66,113	1768	120	57,093	11,392
**Number of packages, n (%)**							
	1		12,235 (17.4)	536 (30.3)	0 (0)	9984 (17.5)	1715 (15.1)
	2		9301 (13.2)	228 (12.9)	5 (4.2)	7968 (14)	1099 (9.6)
	3		7435 (10.6)	113 (6.4)	0 (0)	6300 (11)	1022 (9)
	4		6721 (9.6)	176 (10)	0 (0)	5716 (10)	829 (7.3)
	≥5		34,681 (49.3)	715 (40.4)	115 (95.8)	27,124 (47.5)	6727 (59.1)
**Package sizes, median (IQR)^a^**							
	1		—^b^	30 (30-90)	—	30 (30-100)	30 (30-30)
	2		—	90 (60-90)	—	50 (30-100)	30 (30-30)
	3		—	90 (67.5‐90)	—	50 (30-83)	30 (30-30)
	4		—	90 (90-90)	—	50 (35-72)	30 (30-30)
	≥5		—	90 (90-90)	—	45 (30-60)	30 (30-30)
**Prescriber of the issued prescriptions, n (%)**							
	**General practitioner (GP)**		27,323 (41.3)	639 (36.1)	10 (8.3)	24,127 (42.3)	3529 (31)
		Only	22,578 (82.6)	476 (74.5)	10 (100)	19,521 (80.9)	1762 (49.9)
		>50	1911 (7)	63 (9.9)	0 (0)	1802 (7.5)	717 (20.3)
		Rest	2834 (10.4)	100 (15.6)	0 (0)	2804 (11.6)	1050 (29.8)
	**Psychiatrist**		28,057 (42.4)	336 (19)	5 (4.2)	23,553 (41.3)	6222 (54.6)
		Only	23,267 (82.9)	220 (65.5)	0 (0)	18,167 (77.1)	3204 (51.5)
		>50	2247 (8)	31 (9.2)	5 (100)	2260 (9.6)	1308 (21)
		Rest	2543 (9.1)	85 (25.3)	0 (0)	3126 (13.3)	1710 (27.5)
	**Neurologist**		748 (1.1)	181 (10.2)	37 (30.8)	545 (1)	63 (0.6)
		Only	558 (74.6)	97 (53.6)	11 (29.7)	384 (70.5)	26 (41.3)
		>50	99 (13.2)	31 (17.1)	20 (54.1)	48 (8.8)	25 (39.7)
		Rest	91 (12.2)	53 (29.3)	6 (16.2)	113 (20.7)	12 (19)
	**Other specialists**		7962 (12)	286 (16.2)	15 (12.5)	6680 (11.7)	1300 (11.4)
		Only	4599 (57.8)	176 (61.5)	10 (66.7)	3782 (56.6)	437 (33.6)
		>50	955 (12)	28 (9.8)	5 (33.3)	801 (12)	234 (18)
		Rest	2408 (30.2)	82 (28.7)	0 (0)	2097 (31.4)	629 (48.4)
	**Inpatient psychiatry**		6000 (9.1)	70 (4)	6 (5)	5027 (8.8)	1234 (10.8)
		Only	3429 (57.2)	27 (38.6)	0 (0)	2857 (56.8)	362 (29.3)
		>50	898 (15)	11 (15.7)	0 (0)	756 (15)	214 (17.3)
		Rest	1673 (27.9)	32 (45.7)	6 (100)	1414 (28.1)	658 (53.3)
	**Inpatient acute, rehabilitation, and nursing**		5549 (8.4)	496 (28.1)	60 (50)	4251 (7.4)	1110 (9.7)
		Only	3312 (59.7)	284 (57.3)	11 (18.3)	2376 (55.9)	415 (37.4)
		>50	756 (13.6)	47 (9.5)	32 (53.3)	699 (16.4)	136 (12.3)
		Rest	1481 (26.7)	165 (33.3)	17 (28.3)	1176 (27.7)	559 (50.4)

a Median (IQR) package size (number of units) per number of prescribed packages. Not shown for sodium oxybate, since this substance is a liquid.

b Not applicable.

#### Prescribers

In 2021, psychiatrists were the most frequent prescribers with 42.4% (28,057/66,172) of all the prescribed medications. Lisdexamfetamine (6222/11,396, 54.6%), followed by methylphenidate with 41.3% (23,553/57,038) were mainly prescribed by them. By contrast modafinil and sodium oxybate were rarely prescribed by psychiatrists as 19% and 4.2% (336/1769 and 5/120), respectively. If chosen as a prescriber, psychiatrists are often the only source of prescription for 82.9% (23,267/28,057) insured individuals of all medication of 2021.

The second most frequent prescribers were GPs, with a similar high proportion of 41.3% (27,323/66,172) of all drug prescriptions. More specifically 42.3% (24,127/57,038) methylphenidate was prescribed by GPs followed by modafinil (639/1769, 36.1%), lisdexamfetamine (3529/11,396, 31%), and smaller proportions for sodium oxybate (10/120, 8.3%). GPs were most often the exclusive prescribers of the medications (22,578/27,323, 82.6%). Only 10.4% (2834/27,323) of prescriptions by GPs shared the prescribing job with other medical specialists.

Neurologists were rarely prescribers of stimulants or narcolepsy treatments, as only 1.1% (748/66,172) of all prescriptions were invoiced by them. Only sodium oxybate was the most frequent (37/120, 30.8%) medication prescribed by neurologists. If chosen as the prescriber, they are often the only source (558/748, 74.6%) from which individuals received the prescriptions in 2021 (Table 3).

Concerning invoices handed in by hospitals or psychiatric clinics or rehabilitation facilities, they only made up a small part of overall stimulant invoices. An exception is sodium oxybate, of which 45.1% (60/133) of invoices are issued by an acute clinic, rehabilitation clinic, or nursing home (Table 3).

#### Association of Stimulant Use and Outcomes

We found an association with patients receiving a stimulant or narcolepsy treatment prescription and increased hospitalizations in a psychiatric facility (odds ratio [OR] 5.30, 95% CI 4.63‐6.08, *P*<.001). In contrast, there was a negative association between stimulant prescription and hospitalization in an acute medical care facility (OR 0.77, 95% CI 0.73‐0.82, *P*<.001). There were no significant associations between stimulant prescription and the total length of inpatient stay (OR 1, 95% CI 1‐1, *P*=.13; Table 4).

**Table 4.** Regression model of predicting the outcomes of hospitalization and length of stay in Swiss insureds, who received stimulant prescription in the year 2021.

Characteristic		Odds ratio (95% CI)	*P* value
**Sex**			
	Male	—^a^ (—)	—
	Female	0.89 (0.86‐0.92)	<.001
**Age (years, in groups)**			
	6-17	1.17 (1.13-1.21)	<.001
	18‐35	— (—)	—
	36‐65	0.62 (0.60‐0.64)	<.001
	66‐75	0.31 (0.27‐0.34)	<.001
	76+	0.24 (0.20‐0.28)	<.001
**Region of residence**			
	Urban	— (—)	—
	Intermediate	1.09 (1.05‐1.13)	<.001
	Rural	0.97 (0.94‐1.02)	.2
**Health insurance status**			
	Standard	— (—)	—
	Managed care	0.69 (0.67‐0.71)	<.001
Number of comorbidities		1.56 (1.54‐1.59)	<.001
Total inpatient length of stay		1 (1‐1)	.13
Hospitalization acute (yes or no)		0.77 (0.73‐0.82)	<.001
Hospitalization psychiatry (yes or no)		5.3 (4.63‐6.08)	<.001

a Not applicable.

When only focusing on methylphenidate or lisdexamfetamine compared to a balanced sample of nonstimulant users, we found a positive association between their prescription and hospitalization in a psychiatric facility (OR 6.85, 95% CI 5.89‐7.99, *P*<.001). No significant association was found between the prescription rate and the total inpatient length of stay (OR 1, 95% CI 1‐1, *P*=.1). Hospitalization in an acute medical care facility was less likely (OR 0.76, 95% CI 0.71‐0.8, *P*<.001) to prescribe methylphenidate or lisdexamfetamine (Table 5).

**Table 5.** Regression models of predicting the outcomes of hospitalization and length of stay in Swiss insureds who received stimulant prescriptions in the year 2021. Prescriptions of methylphenidate or lisdexamfetamine are shown in the left columns, prescriptions of modafinil or sodium oxybate are shown in the right columns.

Characteristic		Methylphenidate- or lisdexamfetamine-users versus nonusers		Modafinil- or sodium oxybate-users versus nonusers	
		OR^a^ (95% CI)	*P* value	OR^a^ (95% CI)	*P* value
**Sex**					
	Male	— (—)	—	— (—)	—
	Female	0.9 (0.87‐0.93)	<.001	0.84 (0.72‐0.97)	.02
**Age (years, in groups)**					
	6-17	1.17 (1.13-1.21)	<.001	1.17 (0.73-1.88)	.5
	18‐35	— (—)	—	— (—)	—
	36‐65	0.63 (0.6‐0.65)	<.001	0.57 (0.48‐0.68)	<.001
	66‐75	0.31 (0.28‐0.36)	<.001	0.25 (0.19‐0.34)	<.001
	76+	0.26 (0.21‐0.31)	<.001	0.15 (0.09‐0.23)	<.001
**Region of residence**					
	Urban	— (—)	—	— (—)	—
	Intermediate	1.11 (1.07‐1.15)	<.001	0.96 (0.8‐1.15)	.7
	Rural	0.95 (0.91‐0.99)	.019	1.11 (0.89‐1.38)	.3
**Health insurance status**					
	Standard	— (—)	—	— (—)	—
	Managed care	0.69 (0.67‐0.71)	<.001	0.58 (0.5‐0.68)	<.001
Number of comorbidities		1.55 (1.53‐1.57)	<.001	1.62 (1.54‐1.71)	<.001
Total inpatient length of stay		1 (1-1)	.10	1 (1-1)	.8
Hospitalization acute (yes or no)		0.76 (0.71‐0.8)	<.001	1.23 (0.96‐1.56)	.10
Hospitalization psychiatry (yes or no)		6.85 (5.89‐7.99)	<.001	2.15 (1.17‐4.23)	.02

a Odds ratio

b Not applicable.

When only focusing on modafinil or sodium oxybate compared to a balanced sample of nonstimulant users, we found a positive association between their prescription and hospitalization in a psychiatric facility (OR of 2.15, 95% CI 1.17-4.23, *P*=.02). We found no significant association between total inpatient length of stay (OR 1, 95% CI 1‐1, *P*=.80) and hospitalization in an acute medical care facility (OR 1.23, 95% CI 0.96‐1.56, *P*=.10; Table 5).

## Discussion

### Summary

We found an increasing trend of stimulant and narcoleptic drug prescriptions in Switzerland from 0.6% to 0.8% over the years 2014 to 2021. Most stimulants are prescribed continuously with more than 5 packages in 1 year per insured to underaged individuals with no comorbidities. Psychiatrists and GPs are often the prescribers of stimulants, much more frequently than neurologists.

### Context in Research

Compared with data on global stimulant prescription rates, our results are notably lower, showing a smaller increase in prescription rates than in the United States (5.6%‐6.1%), New Zealand (0.75%‐1.06%), and Denmark (0.03%‐0.73%) [5-7]. Given the ADHDs/ADDs prevalence rates of 4% in adult men and 5.3% in children in Switzerland [2,3], compared to a worldwide prevalence of between 5% and 11.4% [1,15,16], it is reasonable to interpret the lower Swiss prescription rates as either an indication of underprescription or as lower stimulant misuse rates in Switzerland [15]. Even when taking all stimulants together, current prescription rates do not reach the prevalence rate of ADHDs/ADDs in Switzerland. The fact that ADHDs/ADDs are also treated nonpharmacologically is another argument for assuming that our result of low prescription prevalence is lower than the disease prevalence. Unfortunately, there are no data that quantifies the extent of drug or nonpharmacological treatment for ADHDs/ADDs and thus could help define the normal gap between prescription rate and disease prevalence. Our distribution of prescription rates corresponds to the disease distribution in different age groups, with prescriptions and prevalence of ADHDs/ADDs being higher in the younger age groups [17]. The distribution of prescriptions by gender reflects the current disease prevalence of ADHDs/ADDs, as we found a slightly lower proportion of females than males, in line with another summary by Thapar and Cooper [17]

Stimulants were predominantly prescribed in urban areas. A higher population compared to rural areas, a higher density of prescribing physicians, and a higher number of hospitals specialized in diagnosis and treatment of these diseases in urban settings may account for this predominant prescription pattern. Vice versa underprescription in rural areas could be due to reduced access to adequate health care services.

Methylphenidate was by far the most prescribed stimulant agent, followed by lisdexamfetamine, a new stimulant showing promising results for treatment of ADHDs/ADDs [18]. The prescription characteristics were very similar between the two stimulants, with lisdexamfetamine more often prescribed to older adults than methylphenidate. This shows that lisdexamfetamine is possibly used as a second medication after methylphenidate was prescribed in the young and did not give continuous results while the patients aged.

Prescriptions were most frequently issued by psychiatrists. Since methylphenidate and lisdexamfetamine are standard treatment for the psychiatric diseases ADHDs and ADDs, this prescription pattern makes sense from a health provider perspective and is confirmed by other findings about prescribers of stimulants [19].

Prescription characteristics of sodium oxybate should reflect its specificity for narcolepsy because it is not indicated for any other disease. Here we find most of the prescriptions in middle aged groups and fewer in children and teenagers, with a nearly even distribution between women and men. This grouping does not match with the expected narcolepsy features of young patients with a possible second peak in the late forties [20]. The lack of prescription in young patients is best explained by a missed or severely delayed diagnosis of narcolepsy, which is in line with the recently published delayed diagnosis for Switzerland and other European countries [21,22]. Surprisingly, neurologists who diagnose and treat narcolepsy, are rarely the patients’ prescription source, even for highly specific and not easy to handle medication, such as sodium oxybate. Multiple reasons may account for this prescription practice, among them is the lower barrier for receiving an appointment with GPs before prescriptions expires, compared to neurologists.

We found no significant change in the odds of at least one stimulant prescription in patients hospitalized in an acute hospital but a significant increase in the odds of patients hospitalized in a psychiatric facility. This reflects the fact that ADHDs/ADDs are often overlapping or comorbid with other psychiatric diseases which can lead to hospitalization, such as addiction, disruptive disorders, anxiety disorders, or bipolar disorders [23-26], and by the severity of disease.

We assume that our data is representative for Switzerland, since we analyzed claims data extrapolated to the entire population from one of the biggest insurance companies in Switzerland, with a nearly equal distribution across the country. As stimulant agents are only accessed through prescription, we were able to register all invoices for the medication in question in real world; therefore, our data minimize sampling bias and recall bias that frequently influence the accuracy and reliability of retrospective studies.

### Limitations

This study is an analysis of health insurance claims data, which does not contain any information on the clinical reason of why a medication was indicated. We therefore could not distinguish between prescriptions according to current treatment guidelines and prescriptions of pharmacological treatment for diseases without proper diagnosis.

We identified prescribers by Zahlsteller register (registered number for medical personel allowed to bill insurance companies) number and medication prescription pattern, which may lead in some cases to misclassification, as some physicians share Zahlsteller register numbers in group practices and GPs go sometimes through additional training as psychiatrists or neurologists.

### Conclusion

The prescription of stimulants and sodium oxybate in Switzerland increased slightly but continuously over the past years, but at lower rates compared to the estimated prevalence of central hypersomnolence disorders and ADHDs/ADDs. Most stimulants are prescribed by psychiatrists, closely followed by GPs. The increased odds for hospitalization to psychiatric institutions for stimulant receivers reflects the severity of disease and the higher psychiatric comorbidities in these patients.

## Abbreviations

ADHD/ADD: attention-deficit/hyperactivity disorder/attention deficit disorder
GP: general practitioner
OR: odds ratio
